# Evaluation of microbiota-induced changes in biochemical, sensory properties and volatile profile of kombucha produced by reformed microbial community

**DOI:** 10.1016/j.fochx.2024.101469

**Published:** 2024-05-16

**Authors:** Hilal Kilmanoglu, Aycan Yigit Cinar, Muhammed Zeki Durak

**Affiliations:** a Department of Food Processing, Pazarlar Vocational School, Kutahya Dumlupinar University, Kutahya, Türkiye; b Department of Food Engineering, Faculty of Engineering and Natural Sciences, Bursa Technical University, Bursa, Türkiye; c Department of Food Engineering, Faculty of Chemical and Metallurgical Engineering, Yildiz Technical University, Istanbul, Türkiye

**Keywords:** Acetic acid bacteria, Bioactive compounds, Functional beverage, Interaction, Microbial dynamic, Osmophilic yeasts

## Abstract

Kombucha is a traditional beverage produced by a living culture known as SCOBY or “symbiotic culture of bacteria and yeast”. Culture-dependent production is essential for stable kombucha fermentation. The aim of this study was to design a microbial community and to determine the effect of that community on the flavor and chemical properties of kombucha. The fermentations were carried out using combinations of selected species including *Pichia kudriavzevii, Brettanomyces bruxellensis, Dekkera bruxellensis, Komagataeibacter saccharivorans, Komagataeibacter xylinus,* and *Acetobacter papayae*, which were previously isolated from kombucha. The effects of monocultures and cocultures on fermentation were investigated. The highest acetic acid producer was *A. papayae*, which has strong antioxidant properties. In the monoculture and coculture fermentations, aldehydes, acids, and esters were generally observed at the end of fermentation. This study confirms that microbiota reconstruction is a viable approach for achieving the production of kombucha with increased bioactive constituents and consumer acceptance.

## Highlights

• *Brettanomyces bruxellensis* was dominate yeast in kombucha tea.
• *Acetobacter papaya* exhibited the highest acetic acid production.
• The designed consortium produced important flavor and bioactive compounds.
• A consumer-acceptable beverage was performed by microbiota reformed approach.

## 1 Introduction

Kombucha is a low-alcohol (0–1% *v*/v), tea-based fermented beverage with multiple health benefits. It is traditionally obtained by the fermentation of yeasts and acetic acid bacteria embedded in a cellulosic material called SCOBY, or “symbiotic culture of bacteria and yeast”. The traditional beverage is prepared from fermentation of sweetened green or black teas, for 10 to 30 days under aerobic conditions, at 28–29 °C (Amarasinghe, Weerakkody, & Waisundara, 2018; De Filippis, Troise, Vitaglione, & Ercolini, 2018; Laureys, Britton, & De Clippeleer, 2020). While osmophilic yeasts hydrolyze sucrose to glucose and fructose during ethanol fermentation, acetic acid bacteria produce cellulose, acetic acid, and other organic acids (e.g., citric acid, tartaric acid, malonic acid, oxalic acid, succinic acid, and pyruvic acid). Besides the main metabolites of acetic acid and gluconic acid, kombucha also contains various tea compounds (e.g., theaflavin, thearubigin, polyphenols, and catechins), some B complex vitamins, ascorbic acid, carbon dioxide, trace amounts of alcohol, essential minerals, amino acids, biogenic amines, purines, and antibiotics (Dutta & Kr Paul, 2019). Due to the biological activities of these byproducts, which exert antioxidant, antimicrobial, and antidiabetic benefits, the health-promoting effects of kombucha are remarkable (Cardoso, Neto, dos Santos D'Almeida, & do Nascimento, T. P., Pressete, C. G., Azevedo, L., Martino, H. S. D., Cameron, L. C., Ferreirab, M. S. L., & de Barrosa, F. A. R., 2020; Kaewkod, Bovonsombut, & Tragoolpua, 2019; Zubaidah, Afgani, Kalsum, Srianta, & Blanc, 2019). In contrast to the well-known functional beverages based on lactic acid fermentation (such as kefir, boza, and kumiss), studies on kombucha with acetic acid fermentation are gaining popularity.

Previous studies have primarily focused on the microbial composition of SCOBY, which typically includes yeast genera such as *Brettanomyces/Dekkera*, *Zygosaccharomyces*, *Candida*, *Hanseniaspora*, *Torulaspora*, and *Pichia* and bacterial genera such as *Acetobacter*, *Gluconobacter*, *Gluconacetobacter*, and *Komagataeibacter* (Chakravorty et al., 2016; Coton et al., 2017; De Filippis et al., 2018; Laureys et al., 2020; Teoh, Heard, & Cox, 2004).

As with other functional beverages, the composition of kombucha varies according to geographical origin, substrate type, and fermentation conditions (Huang, Xin, & Lu, 2022; Savary et al., 2021). It is also difficult to determine the functional and metabolic behaviour of microorganisms due to the complexity of microbial ecosystems. In this sense, it is necessary to understand the actions of each member of the microbial community to achieve the fermentation of kombucha with a stable chemical composition and appealing sensory properties (Savary et al., 2021). The physicochemical and biochemical properties of kombucha prepared with different microbial strains have been investigated (Ferremi Leali et al., 2022; Tran et al., 2020), but the effects of each strain in different microbial communities on the fermentation properties of kombucha, including the microbial, physicochemical, and volatile components, need to be clearly identified while considering consumer expectations.

The yeast species *Brettanomyces bruxellensis*, *Hanseniaspora valbyensis*, *Zygosaccharomyces bailii*, *Zygosaccharomyces parabailii* and bacterial species *Acetobacter indonesiensis*, *Komagataeibacter hansenii*, *Gluconobacter oxydans*, *Liquorilactobacillus nagelii*, and *Novacetimonas hansenii* were utilized in some previous studies of microbial communities investigating yeast-bacteria interactions for kombucha fermentation (Ferremi Leali et al., 2022; Savary et al., 2021; Tran et al., 2020; Tran et al., 2022). However, studies of yeast-yeast or bacteria-bacteria interactions in kombucha production are not available in the literature.

The first objective of this study was to determine its impact on fermentation of monoculture and coculture (yeast-yeast, bacteria-bacteria, and yeast-bacteria interactions) by selecting the isolated microorganisms from traditional kombucha beverage for reformed microbial community. Then, it was to investigate its impact on fermentation of this community in terms of chemical, microbiological, and volatile compound profiles. As a starter culture, the community responsible for the fermentation closest to the traditional kombucha beverage was lyophilized and its usability in the functional beverage industry was evaluated through sensory analysis.

## 2 Materials and methods

### 2.1 Kombucha fermentation

The fermentation substrate consisted of 100 g/L sugar (Torku, Türkiye), 6 g/L black tea (Lipton, Türkiye), and 100 mL/L previously produced kombucha tea. The SCOBY culture used in fermentation was obtained from three different stores located in Türkiye (Shaman's Secret, Aktaroloji, and Kombucha 2200). The black tea was added to sterilized tap water and infused for 15 min. After the tea leaves were removed, sugar was added and dissolved and the liquid was cooled to room temperature. The fermented tea and SCOBY were added and the mixture was fermented aerobically at 28–30 °C for 14 days (Değirmencioğlu, Yıldız, Sahan, Güldas, & Gürbüz, 2020).

### 2.2 Microbial isolation

Samples (5 g) taken from both the liquid phase and the solid phase of the kombucha fermentation products on days 0, 7, and 14 were mixed in 45 mL of physiological saline solution (0.86% NaCl). Serial dilutions of 1:10 were prepared using the solutions and these dilutions were inoculated on appropriate growth media. The medium for yeasts consisted of Sabouraud agar (#105438, Merck, Germany) and Wallerstein agar (#17222, Merck, Germany), while the medium for bacteria consisted of AAB medium (3% glucose, 0.5% yeast extract, 0.3% peptone, 2% agar, 3% ethanol, and 1% calcium carbonate) and mannitol medium (25 g/L d-mannitol, 5 g/L yeast extract, 3 g/L peptone, and 20 g/L agar). Cultivated petri dishes were incubated at 30 °C for 2–3 days (Teoh, Heard, & Cox, 2004). The colonies collected from the petri dishes (150 bacteria and 150 yeasts) were cultivated in broth media and stored at −40 °C in 40% (*v*/v) glycerol for further analysis.

### 2.3 Microbiota identifying

The colonies were cultivated in 1 mL of broth medium for DNA extraction. Following centrifugation at 13000 rpm for 10 min, pellets were kept at −80 °C for 15 min. After adding 95 μL of 1× TAE (Tris acetate-EDTA) and 5 μL of lysozyme, the microorganisms were incubated at 37 °C for 1 h. Subsequently, 3 μL of proteinase K was added and the microorganisms were left in a water bath at 58 °C for 60 min and then at 95 °C for 8 min. The obtained extracts were stored at −18 °C until polymerase chain reaction (PCR) analysis was performed.

DNA extract (1 μL) was added to 25 μL of PCR mixture (1.5 μL of 25 mM MgCl_2_, 1 μL of 20 mM dNTP mix, 1.25 μL of each primer, 2.5 μL of 10× buffer, and 0.125 μL of Taq polymerase) (Thermo Fisher Scientific, USA). The ITS1 (5’-TCCGTAGGTGAACCTGCGG-3′) and ITS4 (5’-TCCTCCGCTTATTGATATGC-3′) primers (Sentromer, Türkiye) were used for yeasts. The E517F (5’-GCCAGCAGCCGCGGTAA-3′) and E106R (5’-CTCACGRCACGAGCTGACG-3′) primers (Sentromer, Türkiye) were used for bacteria. The thermal cycling conditions for the obtained PCR mixtures (Bio-Rad, Applied Biosystems, USA) of the bacteria and yeasts included 35 or 34 cycles consisting of 5 min at 95 °C or 1 min at 94 °C for preliminary denaturation, 1 min at 94 °C or 30 s at 94 °C for denaturation, 2 min at 55.5 °C or 30 s at 52 °C for annealing, and 2 min at 72 °C or 1 min at 72 °C for the final extension, respectively (Arıkan, Mitchell, Finn, & Gürel, 2020; Tran et al., 2020).

The obtained PCR products were checked via electrophoresis at 120 V for 105 min (Thermo Fisher Scientific, USA) by loading the products into agarose gel wells with concentrations of 1%. Samples that formed the appropriate bands were selected and submitted for sequencing (Medsantek, Türkiye). After sequencing, species were identified using the BLAST tool available via the website of the US National Center for Biotechnology Information. A phylogenetic tree was produced using the MEGA 11 program.

### 2.4 Fermentation trials

Three yeast species and 3 bacteria species were selected from among the identified microorganisms from kombucha. Cultures were grown in the broths in which they were isolated at 28–30 °C for 3 days. The tubes were centrifuged at 4 °C and, 6000 rpm for 10 min and transferred to bottles containing kombucha medium. Fermentations were achieved by the microbial community reconstruction method using yeast-yeast, bacteria-bacteria, and yeast-bacteria interactions at 28–30 °C in kombucha medium (prepared with 200 mL of sterile pure water, 20 g of sucrose, and 1.2 g of black tea as described in Section 2.1). The initial inoculation for isolated cultures was performed at 10^6^ to 10^7^ CFU/mL. Inoculants for the yeast-yeast and bacteria-bacteria combinations were prepared at ratios of 1:1, while inoculants for yeast-bacteria combinations were prepared at ratios of 2:3 (Nguyen, Nguyen, Nguyen, & Le, 2015).

### 2.5 Microbial enumerations

To determine the dynamics of the redesigned microbial communities at days 0, 7, and 14, serial dilutions of 1:10 were prepared and inoculated on agar media. Sabouraud agar was used for yeast counts, AAB medium was used for acetic acid bacteria counts, and mannitol medium was used for gluconobacteria counts. Petri dishes were incubated at 30 °C for 2–3 days. Count results were given as CFU/mL (Neffe-Skocińska, Sionek, Ścibisz, & Kołożyn-Krajewska, 2017).

### 2.6 Determination of total phenolic contents and antioxidant capacity

The DPPH (2,2-diphenyl-1-picrylhydrazyl) method was applied to measure the free radical scavenging activities at each sampling day. Samples of 0.5 mL were mixed with 1.5 mL of 0.1 mM methanolic DPPH solution and incubated in a dark environment at 25 °C for 20 min. The absorbance was then measured at 517 nm with a spectrophotometer (Soif, China). Trolox was used as the standard (1–150 μmol/L) and a calibration curve was produced by measuring absorbance at 517 nm. The results were calculated as mmol Trolox equivalent (TE)/mL (Fu, Yan, Cao, Xie, & Lin, 2014; Villarreal-Soto et al., 2020). Total phenolic contents were determined according to the Folin-Ciocalteu method whereby 0.5 mL of sample was mixed with 1 mL of 7% sodium carbonate. Subsequently, 2 mL of Folin-Ciocalteu reagent was added to the mixture and the mixture was incubated in the dark for 30 min. After incubation, absorbance was measured at 750 nm using a spectrophotometer. Gallic acid calibration curve drawn in the range of 0.1–200 mg/L and results were given as mg gallic acid equivalent (GAE)/L (Değirmencioğlu et al., 2020).

### 2.7 Determination of sugar and acetic acid contents

The sugar and acetic acid contents of the samples were determined by chromatography. Samples of 1 mL or 30 μL filtered through 0.45 μm sterile filters were used for sugar and organic acid analysis, respectively. For sugar analysis, a refractive index (RI) detector with a Hypersil APS-2 column of 100 × 3.0 mm with 5-μm particle size was used. Acetonitrile and ultrapure water (80% ACN/20% H_2_O) were used as the mobile phase, the analysis temperature was 30 °C, and the flow rate was 0.5 mL/min (Neffe-Skocińska et al., 2017). For organic acid analysis, a Zorbax ODS column of 4.6 × 150 mm and a DAD detector (210 nm) were used. For the mobile phase, 0.005 M sulfuric acid was used, while the analysis temperature was 60 °C and the flow rate was 1 mL/min (Jakubcyzk et al., 2020). Sucrose (g/L), glucose (g/L), fructose (g/L), and acetic acid (mg/L) were used as standards in the calibration curves.

### 2.8 Measurements of pH and total titrable acidity

The pH values of the samples were measured with a pH meter (ST300, Ohaus, USA). Total acidity was determined by volumetric analysis method using 0.1 N NaOH solution, 10 mL of sample, and phenolphthalein solution as the indicator, and the results were calculated as g acetic acid/100 mL (Vitas, Cvetanovıć, Maškovıć, Švarc-Gajıć, & Malbaša, 2018).

### 2.9 Degrees brix and optical density (OD) measurements

In order to monitor cell growth, optical density measurements were performed with a spectrophotometer (Soif, China) at 600 nm and the amount of water-soluble dry matter was measured with a refractometer (Isolab, Germany) (Ahmed, Hikal, & Abou-Taleb, 2020).

### 2.10 Determination of volatile compound profiles

Volatile compound analysis was performed by solid-phase microextraction (SPME) using a gas chromatography–mass spectrometry (GC MS-QP2010, Shimadzu, Japan). Samples of 2 mL were placed into SPME vials (Supelco, USA) and kept in a water bath at 60 °C for 15 min to stabilize the volatile compounds in the head space of the vials. An SPME fiber assembly (75 μm, Carboxen/Polydimethylsiloxane) was then immersed into the head space of each vial, which was left at 60 °C for 30 min, allowing the volatile compounds to be collected by the fiber assembly. At the end of this period, the SPME fiber assembly was injected into the GC MS. A Restek Rx-5Sil MS column (30 m × 0.25 mm, 0.25 μm; Restek Corporation, USA) was used with helium (1.61 mL/min) as the carrier gas and the furnace program's initial temperature was 2 min at 40 °C with a final temperature of 250 °C for 5 min. The Wiley, NIST, Tutor, and FFNSC libraries were used to identify volatile compounds (Güneşer et al., 2015; Tu, Tang, Azi, Hu, & Dong, 2019).

### 2.11 Sensory analysis

Sensory analysis was performed with the help of 50 people (32 women and, 18 men, aged 20–50) who had previously consumed kombucha beverage to determine the acceptability and overall quality of the kombucha produced in this study. The samples were randomly coded and presented to the participants, and they were asked to score the samples on a hedonic scale of 7 points based on color, taste, smell, and general acceptability (1 = least acceptable, 7 = most acceptable). In addition, acceptability index values (Eq. 1) were calculated and commercial kombucha and the beverages obtained with the starter culture produced in this study were compared (Kaur, Kaur, Wagh, Kaur, & Aggarwal, 2020; Tu et al., 2019).

(1) $$\mathrm{Acceptability index}\;\left(\%\right)=\left(\mathrm{General acceptability score}/7\right)\;x\;100$$

### 2.12 Lyophilization

The selected strains were cultivated in appropriate liquid media and centrifuged for 10 min at 6000 rpm. Pellets were washed with pure water and mixed with sterile 20% sucrose solution (1:5). Those mixtures were lyophilized after pre-freezing at −40 °C for 2–3 h. Lyophilization was performed in a vacuum of 0.04 mbar at −50 °C. The effectiveness of lyophilization was calculated by the microbial survival rate (Eq. 2) (Sharma et al., 2014). Serial dilutions of lyophilized cultures were prepared in 1:10 physiological saline solution (0.86% NaCl) for this purpose. The yeasts were streaked onto Sabouraud agar and bacteria were streaked onto AAB or mannitol agar and incubated at 28–30 °C for 2–3 days.

(2) $$\mathrm{Microbial survival rate}\;\left(\%\right)=N/N_{0}\;x\;100$$

N = Number of living microorganisms before lyophilization, N_0_ = Number of living microorganisms after lyophilization.

### 2.13 Statistical analysis

The significance of the differences between obtained data (*P* < 0.05) was calculated by Tukey test and one-way analysis of variance (ANOVA). All assays and fermentation trials were repeated three times and data were expressed as means ± standard deviations. For statistical analyses, IBM SPSS Statistics 22 (IBM Corp., USA) was used.

## 3 Results and discussion

### 3.1 Identification of yeast and bacteria isolated from kombucha

At the beginning of the kombucha fermentation, yeast genera such as *Candida*, *Pichia*, *Brettanomyces*, and *Starmerella* were present in liquid phase. Three strains selected from among different yeast species isolated on Wallerstein and Sabouraud agar had different morphological characteristics. On Wallerstein agar, *Pichia kudriavzevii* formed dark green colonies, *Brettanomyces bruxellensis* formed white colonies, and *Dekkera bruxellensis* formed light green-yellow colonies. The selection of acetic acid bacteria was determined based on the formation of a clear zone (*Acetobacter papayae*) upon dissolving CaCO_3_ on AAB agar, and by the formation of transparent white colonies (*Komagataeibacter xylinus*) and soft white colonies (*Komagataeibacter saccharivorans*) on mannitol agar. In addition, agarose gel images of selected yeasts and bacteria and their nucleotide sequences after sequencing process are presented in Fig. S1 and Table S1, respectively.

**Fig. 1:**
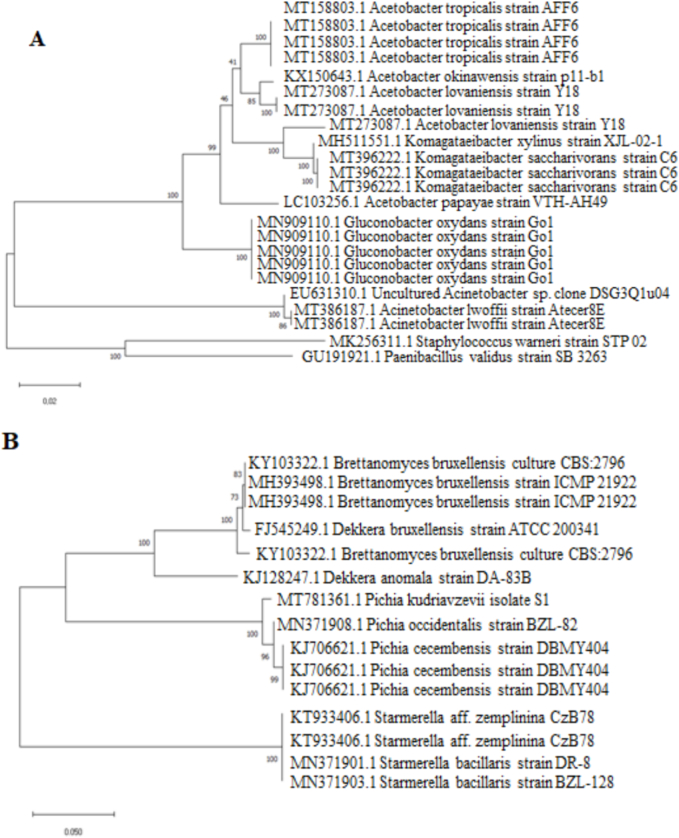
Dendrogram showing the clustering bacteria (A) and yeasts (B).

However, as fermentation progressed, this variety decreased and *Pichia* and *Brettanomyces* became dominant in the environment (Fig. 1). Since *Brettanomyces* species are resistant to high acidity and have the ability to produce acetic acid, it is to be expected that this genus constitutes the dominant yeast species in kombucha (Chakravorty et al., 2016; Coton et al., 2017; De Filippis et al., 2018).

The dominant bacterial genera found in the kombucha at the end of 14 days of fermentation were *Acetobacter* and *Gluconobacter*. Besides these, *Komagataeibacter*, *Gluconacetobacter*, and *Paenibacillus* were also noted in the literature as bacterial genera found in kombucha. The presence of lactic acid bacteria (LAB) in kombucha is variable. Usually, they are absent or present at low levels. Kombucha's fermentation temperature is slightly lower for LAB and they grow in relatively anaerobic conditions (Coton et al., 2017; Kieliszek, Pobiega, Piwowarek, & Kot, 2021; Laureys et al., 2020). In the present study, these genera were usually isolated at the beginning of fermentation. Acetic acid, which is one of the most important metabolites for kombucha, is produced by *Acetobacter* species oxidizing ethanol. Thus, the presence of acetic acid bacteria in kombucha is important.

### 3.2 Fermentation characteristics of yeasts and bacteria

Three yeasts and three bacteria were selected for analysis because they produced high levels of acidity and survived the fermentation process in addition to possessing functional properties such as high phenolic contents and antioxidant capacity (Li et al., 2022). From among yeasts, *Pichia kudriavzevii* (Pk), *Brettanomyces bruxellensis* culture CBS:2796 (Bb), and *Dekkera bruxellensis* strain ATCC 200341 (Db) were selected, while from among bacteria, *Acetobacter papayae* VTH-AH49 (Ap), *Komagataeibacter xylinus* strain XJL-02-1 (Kx), and *Komagataeibacter saccharivorans* strain C6 (Ks) were selected.

Changes in biochemical profile during monoculture fermentations were assessed as presented in Fig. S2 of the supplementary material. At the end of fermentation, the average pH values for all fermentations decreased significantly (*P* < 0.05). The fermentations of Kx and Ks showed a slight decline after 14 days. The highest level of titrable acidity was demonstrated in yeast's fermentations. Titrable acidity, which was initially 0.01%, exceeded 1% in yeast fermentations, while it remained statistically significantly lower in bacterial fermentations, except for Ap. The genus *Dekkera/Brettanomyces* are known to be well adapted to the nutrient-poor environment (Grassi et al., 2022).

While there was a significant increase in the OD of all fermentations except Ks during the first 7 days of fermentation (*P* < 0.05), this increase slowed toward the end of the fermentation. Furthermore, microbial counts reached their highest values on day 7 of the fermentations and then progressively decreased (Fig. S3). Comparison of the monoculture growth dynamics and OD revealed that the yeast was more dominant than the bacterium. This is because yeasts are more resistant to metabolites such as acid and ethanol. Yeasts are able to metabolize sucrose to glucose and fructose, increasing ethanol and biomass. Acetic acid bacteria can use sucrose as a carbon source, but their metabolic activity is accelerated in the presence of glucose and ethanol (Huang et al., 2022; Li et al., 2022).

It was also observed that there was a decrease in total phenolic contents in all monoculture fermentations, except Ap, on day 7 of fermentation, while total phenolic contents increased at different rates over the following days of fermentation. The decrease in total phenolic contents, which were initially 645 mg GAE/L, in the first 7 days of fermentation was likely a result of the rapid increase in acidity due to microbial activities and the subsequent breakdown of phenolic compounds (Amarasinghe et al., 2018). The total phenolic contents increased at the end of the fermentation in all groups, except for Pk and Ks. Antioxidant capacity analyses revealed that there was an increase in antioxidant capacity in all groups as fermentation progressed, and this increase was statistically significant (P < 0.05), except for Bb. Bacteria may have higher bioactivity due to enzymatic degradation or yeasts that metabolize phenolic compounds to produce other compounds (Zhou et al., 2022).

The decrease in degrees Brix was observed to end at day 7 for the bacteria fermentations, while it continued in yeast fermentations, because these strains utilized different amount of sugars (Jakubczyk, Gutowska, Antoniewicz, & Janda, 2020). The changes in sugar profiles during the fermentations are shown in Table S2 of the supplementary material. It was observed that sugar consumption by yeasts was much higher compared to bacteria. This indicates that yeasts are more active at the beginning of kombucha fermentation and are better able to break down sucrose thanks to their invertase activity (Tran et al., 2020). At the end of the fermentations, Bb and Ap had the highest total rates of sucrose consumption. Acetic acid was produced by all strains, except for Pk. It is known that yeasts of the genera *Brettanomyces* and *Dekkera* in particular produce high amounts of ethanol and acetic acid, while Pk produces high amounts of alcohols (Agnolucci, Tirelli, Cocolin, & Toffanin, 2017).

### 3.3 Characteristics of yeast-yeast fermentations

The counts of all yeasts significantly increased until day 7 of fermentation, except for Db, and continued to multiply until the end (Fig. S4). Although *Dekkera* and *Brettanomyces* belong to the same taxonomic family (Pichiaceae), they have important differences, such as their reproductive forms. In addition, Bb is more tolerant to ethanol and high levels of acidity than *Dekkera* spp. This may be the reason why the counts of *Dekkera* were decreased at day 7 of fermentation (Agnolucci et al., 2017).

**Fig. 2:**
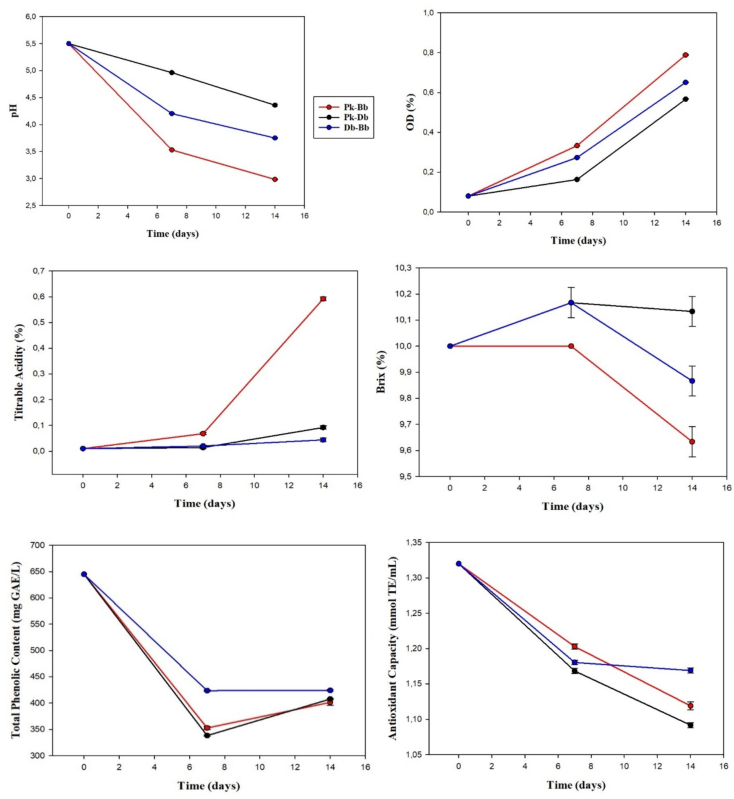
Chemical analysis results at different times of yeast-yeast fermentations (Different letters indicate significant difference in end and start of fermentation, P < 0.05, Pk: *Pichia kudriavzevii*, Bb: *Brettanomyces bruxellensis*, Db: *Dekkera bruxellensis*).

Parameters used to evaluate the symbiotic relationships of yeasts (pH, titrable acidity, Brix, optical density, total phenolic contents, and antioxidant capacity) were also measured (Fig. 2). When the initial and final pH values and titrable acidity of the fermentations were compared, it was observed that pH decreased and titrable acidity significantly increased in all cases. Total acidity increased from approximately 0.01% to 0.6% while pH decreased from 5.5 to 3 with the Bb - Pk coculture. During the fermentations, Brix values significantly decreased and optical density increased (*P* < 0.05). Yeast strains have different tolerances to increasing ethanol and decreasing oxygen as a result of alcohol fermentation (Agnolucci et al., 2017). Bb continued to grow and produce organic acid and attracted attention among other yeast strains.

The total phenolic contents had significantly decreased at the end of all fermentations compared to the initial values. Similar results were obtained for antioxidant capacity. Total phenolic content and antioxidant capacity increased at the end of the mono-yeast fermentations, except for Bb, but decreases in total phenolic content and antioxidant capacity were obtained for yeast-yeast fermentations. Phenolic compounds can be formed as a result of yeast metabolism during fermentation, or phenolic compounds can be released by the presence of various enzymes. The reason for the decrease in the amount of phenolic substances in yeast-yeast fermentations may be nutrient competition. Phenolic compounds in black tea or bioactive substances produced by yeasts may be used as carbon or nitrogen sources and their amount may decrease when sucrose used as a carbon source decreases (Zhou et al., 2022). The mono- and coculture interactions of Pk in kombucha medium have not been previously investigated. When the fermentations were completed, a large amount of the initial sucrose was hydrolyzed by Bb - Pk (Table S3). Additionally, acetic acid was only produced by Bb - Pk. Since the acetic acid formed in mono-yeast fermentations could not be achieved in the other yeast-yeast fermentations, yeast communities negatively affected the production of acetic acid. Overall, it was seen that yeast cocultures did not produce positive symbiotic relationships. Likely due to nutrient competition, the yeasts were unable to produce the desired metabolites.

### 3.4 Characteristics of bacteria-bacteria fermentations

**Fig. 3:**
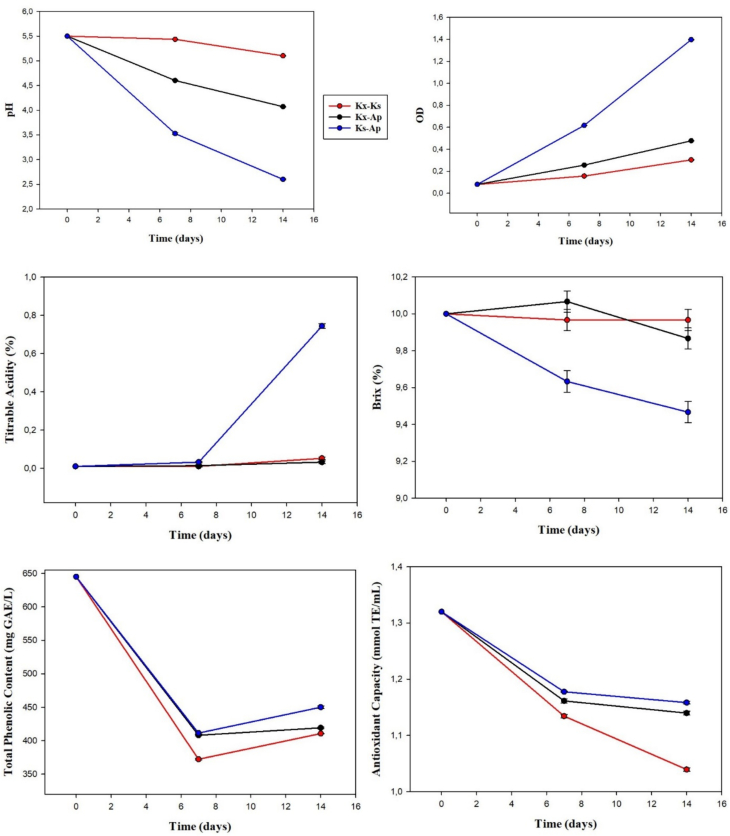
Chemical analysis results at different times of bacteria-bacteria fermentations (Different letters show significant difference in end and start of fermentation, P < 0.05, Ap: *Acetobacter papayae*, Kx: *Komagataeibacter xylinus*, Ks: *Komagataeibacter saccharivorans*).

In fermentations with bacteria communities, pH values decreased and total acidity increased as fermentation progressed (Fig. 3). pH decreased from an initial value of 5.5 to nearly 2 at the end of fermentation by Ap - Ks. At the same time, total acidity increased significantly (*P* < 0.05), reaching 0.7–0.8% at the end of fermentation. It was previously reported that species of *Komagataeibacter* use sugars for cellulose production and produce low levels of organic acids It is also noted that increased acidity has a negative effect on biocellulose production (Gopu & Govindan, 2018). Changes in degrees Brix and optical density values at the end of fermentation were statistically significant (P < 0.05) depending on sugar consumption and microbial proliferation (Fig. 3). Compared to bacterial monocultures, total phenolic contents and antioxidant capacity were decreased with bacteria-bacteria fermentations (Fig. 3). Due to competition, bacteria may utilise tea polyphenols in their metabolic activities and in this case bioactive compounds may not be obtained as much as in mono-bacteria fermentations (Neffe-Skocińska et al., 2017).

In all fermentations, microbial counts had significantly increased at the end of 14 days (Fig. S5). The counts of Ks - Kx coculture increased as fermentation progressed, but when each of those strains was combined with Ap, their counts decreased after day 7 of fermentation. On the other hand, Ap counts in coculture increased until the end of fermentation. It has been reported that the genus *Acetobacter* has a higher tolerance to high acidity that *Komagataeibacter* (Gopu & Govindan, 2018).

The highest levels of sucrose hydrolysis were obtained from Ap - Ks fermentation, while the lowest were observed for Ks - Kx fermentation (Table S4). Acetic acid production was lower in bacteria-bacteria fermentations compared to fermentations with bacterial monocultures. Restricted access to glucose may lead to a reduction in the production of organic acids and the use of organic acids as an energy source.

### 3.5 Characteristics of yeast-bacteria fermentations

Depending on the microflora, but also on the fermentation parameters (type and amount of substrate, inoculation rate, duration, temperature), the presence and amount of chemical compounds in kombucha vary (Bishop et al., 2022). When comparing green, oolong and black tea kombuchas, the amount of acetic acid and antioxidant activities vary due to the different oxidation levels of phenolic substances. It is noted that green tea kombucha contains the highest amount of these substances (Kaewkod et al., 2019). The use of substances such as molasses and fructose instead of sucrose as a carbon source also results in differences in the amount of bioactive compounds in kombucha. Kombucha made from molasses was found to produce more biomass and lactic acid, while kombucha made from sucrose produced more acetic acid (Malbasa, Loncar, Djuric, & Dosenovic, 2008). The high acidity of molasses probably had a negative effect on the growth of acetic acid bacteria and reduced the production of acetic acid. The presence of so many fermentation parameters and especially the complex microbiota makes it difficult for bioactive compounds to remain stable. Therefore, based on some chemical and physical properties of kombucha, different coculture fermentations were carried out to obtain the beverage most similar to traditional kombucha.

In commercial traditional kombucha fermentations, pH values significantly decreased from 3.96 ± 0.05 to 2.29 ± 0.18 and titrable acidity significantly increased from 0.05 ± 0.03% to 1.02 ± 0.14%. Compared to all coculture fermentations, the pH drop in the commercial kombucha was smaller, probably due to the product's higher buffering capacity. Tran et al. (2020) reported that the decreases in pH and total acidity values achieved in kombucha fermentation with Ks were larger when that strain was cocultured with *Saccharomyces cerevisiae* and Bb compared to coculture fermentations with *Acetobacter indonesiensis* and Ap*.*

**Fig. 4:**
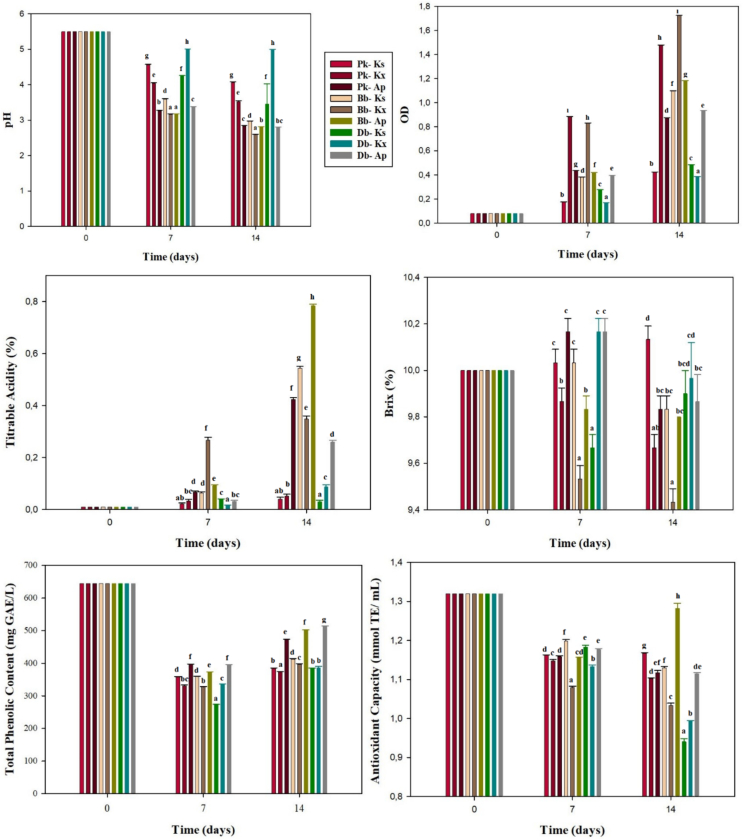
Chemical analysis results at different times of yeast-bacteria fermentations (Different letters show significant difference in same day of fermentation, P < 0.05, Pk: *Pichia kudriavzevii*, Bb: *Brettanomyces bruxellensis*, Db: *Dekkera bruxellensis*, Ap: *Acetobacter papayae*, Kx: *Komagataeibacter xylinus*, Ks: *Komagataeibacter saccharivorans*).

In the present study, in fermentations including Ap, a significant decrease of pH was observed and the highest titrable acidity was obtained by Bb - Ap (Fig. 4). Although high optical density and low Brix values were obtained in fermentations containing Kx and Ks, the total acidity remained low, probably due to the use of sugars during the production of cellulose (Gopu & Govindan, 2018). Total phenolic contents significantly decreased in all fermentations. The total phenolic contents of the commercial kombucha at day 14 were 416.77 ± 8.95 mg GAE/L while total antioxidant capacity was 1.22 ± 0.01 mmol TE/mL. The polyphenol contents produced by Ap - yeast cocultures were greater compared to commercial kombucha (*P* < 0.05).

Bioactive components such as acetic acid and phenolic compounds in kombucha beverage not only protect it from pathogenic microorganisms, but also promote health. This property is attributed to the antioxidant capacity of phenolic compounds such as catechin. Lactic acid fermented beverages (kefir, ayran) are also noteworthy among fermented beverages with beneficial effects on human health. The antioxidant capacity of fermented milk is reported to be about 0.16–1.03 mmol TE/mL (Stobiecka, Król, & Brodziak, 2022). The highest antioxidant capacity of coculture fermentations was obtained with Bb - Ap (1.3–0.01 mmol TE/mL). This value varies because the type and concentration of tea used, fermentation time and microbial composition significantly affect the antioxidant properties.

In kombucha prepared with black tea, an antioxidant capacity of approximately 10 mmol TE/mL was determined after 8 weeks of fermentation (Amarasinghe et al., 2018).

**Fig. 5:**
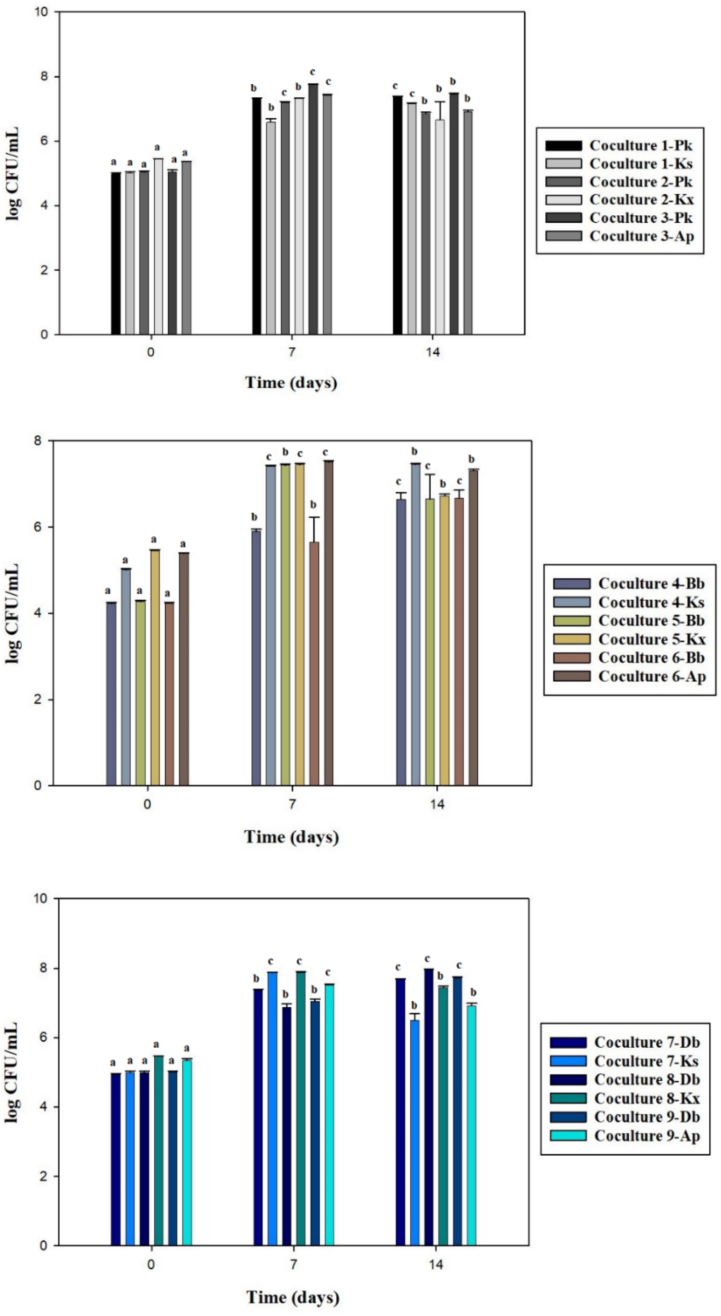
Microbial counts of yeast-bacteria fermentations (Different letters show significant difference on different fermentation days of a strain, P < 0.05, Pk: *Pichia kudriavzevii*, Bb: *Brettanomyces bruxellensis*, Db: *Dekkera bruxellensis*, Ap: *Acetobacter papayae*, Kx: *Komagataeibacter xylinus*, Ks: *Komagataeibacter saccharivorans*).

It may be concluded that higher initial counts of bacteria were more important compared to initial yeast counts producing the desired final product. Yeasts develop much faster than bacteria, which can cause yeasts to inhibit bacteria, thus lowering the production of the expected metabolites. Therefore, yeast-bacteria fermentations were begun at yeast-to-bacteria ratios of 2:3 (Nguyen et al., 2015). In addition, when yeast and bacteria are inoculated at the same rate, the number of acetic acid bacteria decreases due to restricted oxygen access (Tran et al., 2022). Yeast counts were higher than bacteria counts on day 14 for all fermentations except Bb - Ks and Bb - Ap (Fig. 5). The counts of the monocultures at day 14 were lower than those of the cocultures. In a similar study, population counts of *Acetobacter* spp. were higher in monocultures, while counts of Ks were higher in cocultures. It was also reported that total yeast and bacterial population counts in the original kombucha fermentation were higher than those of yeast - bacteria fermentations (Tran et al., 2020). In the present study, in commercial kombucha fermentation at day 14, total yeast and bacteria counts had increased by approximately 2 log units, reaching 6.54 ± 0.10 log CFU/mL and 5.94 ± 0.30 log CFU/mL, respectively. Compared to yeast-bacteria fermentations, commercial kombuchas have higher microbial population counts. This difference could be explained by inhibition through the production of antiproliferative substances such as acetic acid and nutritional competition (Tran et al., 2020).

**Fig. 6:**
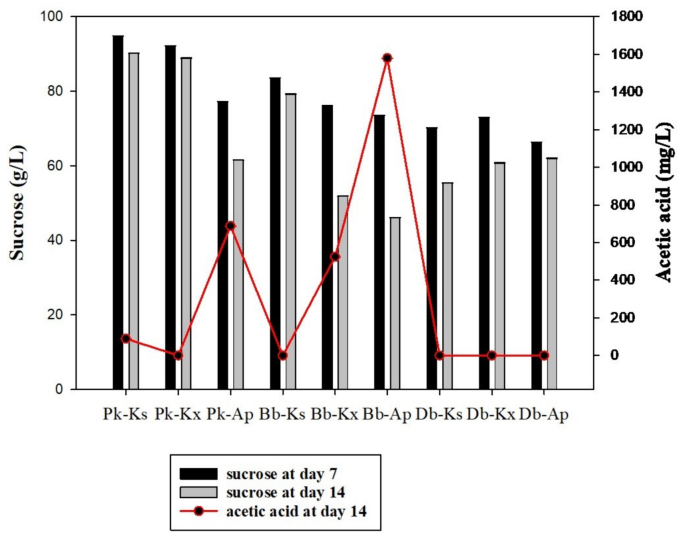
The amounts of sucrose and acetic acid determined in yeast-bacteria fermentations (All of communities showed significant difference in same day of fermentation, *P* < 0.05, Pk: *Pichia kudriavzevii*, Bb: *Brettanomyces bruxellensis*, Db: *Dekkera bruxellensis*, Ap: *Acetobacter papayae*, Kx: *Komagataeibacter xylinus*, Ks: *Komagataeibacter saccharivorans*).

Total sucrose values differed significantly among the yeast-bacteria fermentations (Fig. 6). Monoculture fermentations had higher values of sucrose hydrolysis compared to coculture fermentations. This reflects the invertase activity of yeasts. Bb - Ap achieved significantly stronger degradation of sucrose compared to other cocultures (*P* < 0.05).

In combinations with Pk, Ks, and Kx, most of the sucrose was not utilized during fermentation. The fructose contents of the cocultures were found to be higher than the glucose contents, except for Db - Ap and Bb - Ks (Table 1). This may suggest that the acetic acid bacteria prefer glucose. Sucrose was completely depleted in the commercial kombucha fermentation over the course of 14 days, while the glucose and fructose contents reached values of 32 ± 0.22 g/L and 14.03 ± 0.06 g/L, respectively. It can be said that sugar utilization was high in this kombucha due to the complex microbial diversity of commercial SCOBY.

**Table 1:** The amount of glucose and fructose produced by yeast-bacteria fermentations (mean ± standard deviation).

Strains	Glucose (g/L)			Fructose (g/L)		
	t_0_	t_7_	t_14_	t_0_	t_7_	t_14_
Pk-Ks	0 ± 0.0	2.04 ± 0.02^b^	1.76 ± 0.02^d^	0 ± 0.0	3.33 ± 0.02^d^	2.14 ± 0.02^e^
Pk-Kx	0 ± 0.0	2.72 ± 0.02^c^	1.56 ± 0.01^c^	0 ± 0.0	2.99 ± 0.01^b^	2.04 ± 0.01^d^
Pk-Ap	0 ± 0.0	2.98 ± 0.01^d^	0.15 ± 0.01^a^	0 ± 0.0	3.05 ± 0.01^c^	1.26 ± 0.02^b^
Bb-Ks	0 ± 0.0	1.87 ± 0.01^a^	0.46 ± 0.01^b^	0 ± 0.0	1.89 ± 0.01^a^	0.34 ± 0.02^a^
Bb-Kx	0 ± 0.0	5.32 ± 0.02^e^	2.14 ± 0.02^f^	0 ± 0.0	4.86 ± 0.02^e^	2.59 ± 0.01^f^
Bb-Ap	0 ± 0.0	6.64 ± 0.02^f^	5.06 ± 0.01^h^	0 ± 0.0	7.04 ± 0.02^f^	6.13 ± 0.02^ı^
Db-Ks	0 ± 0.0	7.77 ± 0.02^g^	1.87 ± 0.01^e^	0 ± 0.0	8.43 ± 0.01^h^	3.74 ± 0.01^g^
Db-Kx	0 ± 0.0	7.42 ± 0.01^h^	2.15 ± 0.01^fg^	0 ± 0.0	8.13 ± 0.01^g^	3.88 ± 0.01^h^
Db-Ap	0 ± 0.0	10.18 ± 0.01^ı^	2.19 ± 0.01^g^	0 ± 0.0	10.37 ± 0.01^ı^	1.86 ± 0.01^c^

The highest acetic acid level was achieved by Bb-Ap, while no acetic acid was detected in fermentations with Db (Fig. 6). Interestingly, although *Brettanomyces* and *Dekkera* species are known to produce acetic acid, no acetic acid was detected in fermentations containing Db. The increased microbial population in the fermentation may have restricted access to oxygen, preventing the production of ethanol and thus preventing acetic acid production by the bacteria (Agnolucci et al., 2017). The amount of acetic acid determined in the commercial kombucha was about 5.6 times higher than the highest amount of acetic acid achieved among the yeast-bacteria cultures. Tran et al. (2020) detected 2 g/L acetic acid in original kombucha samples. Their result is close to the amount of acetic acid obtained from Bb - Ap fermentation in the present study.

t_0_: Fermentatiton day 0, t_7_: Fermentatiton day 7, t_14_: Fermentatiton day 14, different letters in the same column show significant differences (P < 0.05) according to the Tukey test.

### 3.6 Volatile compounds produced in fermentations

The 30 volatile compounds detected in this study, excluding hydrocarbon compounds, included alcohols, aldehydes, ketones, terpenes, acids, and esters (Table 2). The volatile compound profile of unfermented tea consists of aldehydes or saturated hydrocarbons (Kraujalyte, Pelvan, & Alasalvar, 2016).

**Table 2:** Volatile compounds determined in fermented and unfermented sweet tea medium.

Retention time	IUPAC name	Common name	Chemical group	Fermentation where it was detected
1.283	Ethanal	Acetaldehyde	Aldehyde	Db-Ks, Pk, Kombucha, Ks-Kx, Ks-Ap
1.375	Ethanol	Ethanol	Primary alcohol	Kombucha, Ks-Kx, Ks, Pk-Bb, Pk-Ks, Bb-Ap, Db-Ks
1.450	Propan-2-one	2-propanone	Ketone	Bb-Ks, Ks, Ap-Kx
1.726	2-methylbutanal	2-methylbutanal	Aldehyde	Bb-Ks, Pk, Tea broth, Db, Db-Bb, Db-Pk, Db-Kx, Bb
1.916	Ethyl acetate	Ethyl acetate	Ester	Tea broth, Db-Ks, Pk-Bb, Pk-Ap, Bb-Ap, Pk-Ks, Kombucha, Ks-Kx
2.067	Acetic acid	Acetic acid	Acid	Ap, Kombucha, Ap-Ks, Pk-Ap, Bb-Ap, Bb-Kx, Bb-Pk, Db-Ap
2.700	Pentan-3-one	3-pentanone	Ketone	Pk-Db, Db-Kx, Ap, Pk
2.706	Pentanal	Pentanal	Aldehyde	Kombucha
2.900	3-hydroxybutan-2-one	Acetoin	Ketone	Db-Ks
3.343	3-methylbutan-1-ol	Isoamyl alcohol	Primary alcohol	Db-Ks, Bb-Ks, Ap-Pk, Ap-Bb, Kombucha, Ap-Ks, Ap-Kx, Ks-Kx
3.405	2-methylbutan-1-ol	2-methyl-1-butanol	Primary alcohol	Ap-Kx, Ks-Kx, Pk-Bb, Db-Ks, Pk-Ap
3.871	2-methylpropanoic acid	Isobutiric acid	Fatty acid	Pk-Bb, Bb-Kx, Ks-Kx
4.023	2-methylpropyl acetate	Isobutyl acetate	Ester	Db-Ks
6.147	Hex-2-enal	trans-2-hexenal	Aldehyde	Kombucha
6.315	3-Methylbutanoic acid	Isovaleric acid	Fatty acid	Pk-Bb, Pk-Ap, Bb-Kx
6.559	2-Methyl butanoic acid	2-Methyl butanoic acid	Conjugated acid	Ks-Kx, Ap-Ks, Pk-Bb, Bb-Kx, Db-Ks
6.888	3-methylbutyl acetate	Isoamyl acetate	Ester	Db-Ks
9.894	Benzaldehyde	Benzaldehyde	Aldehyde	Db-Ks
10.987	7-methyl-3-methylideneocta-1,6-diene	beta-Myrcene	Terpene	Ks-Kx
11.520	Octanal	Octanal	Fat aldehyde	Kombucha
11.571	2-methyl-5-propan-2-ylcyclohexa-1,3-diene	Alpha-phellandrene	Terpene	Ks-Kx
11.666	1-methyl-4-propan-2-ylcyclohexa-1,4-diene	Gamma-terpınene	Terpene	Ks-Kx
12.480	1-methyl-4-prop-1-en-2-ylcyclohexene	Lımonene	Terpene	Ks-Kx
12.600	2-ethylhexan-1-ol	2-Ethyl hexanol	Primary alcohol	Db, Pk-Db, Db-Kx, Pk, Pk-Bb, Kombucha, Ap
15.341	3,7-dimethylocta-1,6-dien-3-ol	Linalool	Tertiary alcohol	Kombucha, Ks-Kx, Pk, Pk-Bb, Pk-Ks, Bb-Kx, Tea broth, Bb
15.466	Nonanal	Nonanal	Fat aldehyde	Kombucha
15.742	2-phenylethanol	Phenylethyl Alcohol	Primary alcohol	Db-Ks
29.776	2,4-Di-tert-butylphenol	2,4-Di-tert-butylphenol	Phenol	Pk, Ap

Tran et al. (2022) identified the volatile compounds produced during kombucha fermentation by two yeasts isolated from kombucha (*B. bruxellensis* and *H. valbyensis*) and reported that saturated fatty acids were the signature metabolites of those yeasts. However, in the present study, those fatty acids could not be detected in any of the Bb fermentations. As a result of the accumulation of carbohydrates, the growth of some microorganisms is prevented and the production of fatty acids may be reduced or delayed as a result of the inability to use nitrogenous compounds. While volatile components were produced by Ap such as acetic acid, ketones, and alcohols, the volatile compound profiles of the two other bacterial strains were very limited. The richness of aroma compounds in kombucha is associated with the completion of its fermentation (Wang et al., 2022). The interaction of fatty acids (acetic acid, isovaleric acid) and alcohols (isobutanol, phenylethyl alcohol) in kombucha is known to influence sensory properties. The interaction of fatty acids (acetic acid, isovaleric acid) and alcohols (isobutanol, phenylethyl alcohol) in kombucha is known to influence sensory properties. The presence of these compounds contributes to the fruity and vinegary flavours in the beverage. In particular, isovaleric acid and phenyl ethyl alcohol were only formed in yeast - bacteria fermentations. This suggests that microbial diversity influences volatile compounds and therefore, can influence consumer taste by altering the sensory properties of the product (Tran et al., 2022). Acetic acid, as one of the important metabolites found in kombucha, was clearly observed in cocultures with Ap. Alcohols and esters, which have significant sensory effects on fermented products, are commonly known as yeasts' key metabolites (Tran et al., 2022). Such metabolites were associated with Db in the present study. However, considering the ideal physicochemical properties of kombucha, the cocultures with Db were insufficient.

Based on these analyses, the Bb - Ap coculture was selected in terms of the desired properties of the final product and the presence of all evaluated volatile compounds (Cardoso et al., 2020; Savary et al., 2021; Tran et al., 2022; Tu et al., 2019).

After the lyophilization of Bb - Ap, the count of the yeast decreased from 1.2 × 10^8^ to 1.9 × 10^7^ CFU/mL, while the count of bacteria decreased from 5.5 × 10^7^ to 4 × 10^6^ CFU/mL. In other words, cell bioavailability was 95% for yeasts and 90% for bacteria, which are markedly high levels. It has been reported that various carbohydrate and protein-based substances used as protective agents in lyophilization can increase the survival rates of microorganisms by up to 96% on average (Sharma et al., 2004). It has also been reported that in spray drying, another drying method used in starter culture production, this rate of increase is approximately 83–93% (Hamsupo, Sukyai, Nitisinprasert, & Wanchaitanawong, 2005).

### 3.7 Sensory analysis

Sensory analysis was conducted to evaluate both the reconstructed microbial community and the commercial kombucha. The taste score for the commercial kombucha was 5.2 ± 0.51, which was 9.5% lower than the score obtained for the beverage produced with Bb - Ap and 8% lower than the score for the beverage produced with lyophilized Bb - Ap (Fig. S6). Similarly, the odor score of the commercial kombucha was 3.85 ± 0.65, which was 24% lower than the score for the beverage produced with Bb - Ap and 26% lower than the score for the beverage produced with lyophilized Bb - Ap. Commercial kombucha has much higher microbial diversity; thus, it is likely to have higher acidity and a more sour taste and smell due to the presence of various organic acids (Treviño-Garza et al., 2020; Zou et al., 2021). For this reason, the beverages produced with Bb - Ap were determined to be more acceptable in terms of taste and smell. However, the three beverages had very close color scores, with commercial kombucha having the highest color score of 5.35 ± 0.65. When the general acceptability scores and index values were examined, the beverage produced with Bb - Ap was found to have the highest scores in both cases. The results for general acceptability and the acceptability index did not differ significantly between the commercial kombucha and the beverage produced with a reconstructed microbial community. These results suggest that the lyophilized Bb - Ap community could serve as an appropriate kombucha starter.

## 4 Conclusion

Kombucha has many valuable components and biological activities which may have a potential impact on human health. The complex yeast-bacteria consortium utilizes substrates in various pathways to produce several metabolites. The aim of this study was to create a starter culture for traditional kombucha beverage, which do not have specific compositions and have varying proportions and types of metabolites in each production batch. As a consequence of fermentation trials, cocultures of yeast-yeast and bacteria-bacteria did not yield positive synergistic relationships. However, the yeast-bacteria cocultures provided the possibility of standardized kombucha production. The results most similar to the characteristics of commercial kombucha were obtained with Bb - Ap. As a result of kombucha fermentations performed with commercial SCOBY, the pH was 2.29 ± 0.18, total acidity was 1.02 ± 0.14%, total phenolic contents were 416.77 ± 8.95 mg GAE/mL, and antioxidant capacity was 1.22 ± 0.01 mmol TE/mL.

The sensory results of the beverage produced using Bb - Ap also support the potential value of the lyophilized coculture. The ability of symbiotic relationships involving at least one yeast and one acetic acid bacterial strain to produce a beverage similar to traditional kombucha was revealed in terms of the sensory results, volatile component profile, and chemical properties. This study has provided a novel perspective on the applicability of a reconstructed microbial community for kombucha fermentation. Once the microbial capacity has been established, further research can focus on the standardization of other parameters that influence kombucha characteristics (e.g. fermentation time, temperature, substrate quantity, inoculum size) with descriptive analysis and preference mapping approaches.

## Ethical statement

Ethical permission, to conduct a human sensory study, is not customary or a requirement of your institution. However, all authors confirm that the rights and privacy of all participants were protected during the execution of the research, e.g., no coercion to participate, full disclosure of study requirements and risks, written or verbal consent of participants, no release of participant data without their knowledge, ability to withdraw from the study at any time.

## CRediT authorship contribution statement

**Hilal Kilmanoglu:** Writing – review & editing, Writing – original draft, Methodology, Investigation, Conceptualization. **Aycan Yigit Cinar:** Writing – review & editing, Writing – original draft, Methodology, Investigation, Conceptualization. **Muhammed Zeki Durak:** Writing – review & editing, Methodology, Investigation, Conceptualization.

## Declaration of competing ınterest

The authors declare that they have no known competing financial interests or personal relationships that could have appeared to influence the work reported in this paper.

## Data Availability

Data will be made available on request.

## Appendix A Supplementary data

Supplementary material. Image 1
