# Supplementary material for: Evaluation of microbiota-induced changes in biochemical, sensory properties and volatile profile of kombucha produced by reformed microbial community
Source: Food Chem X. 2024 May 16;22:101469. doi: 10.1016/j.fochx.2024.101469 (PMC11130685; doi:10.1016/j.fochx.2024.101469)
Supplement: Supplementary file 1 — Supplementary material. [file mmc1.docx]

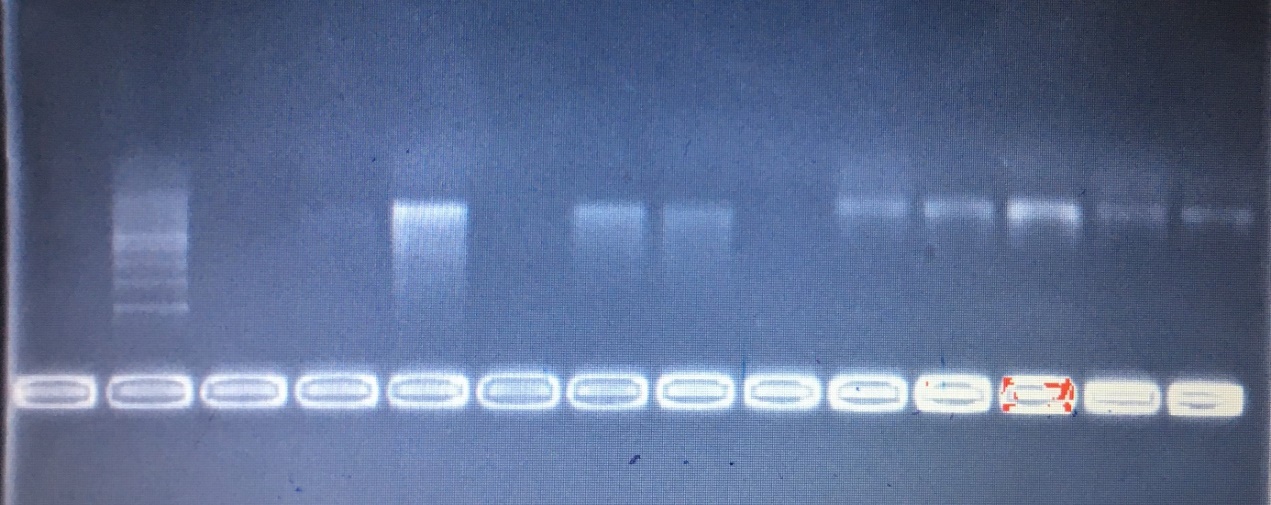


**M**

**Ap**

**Kx**

**Ks**

**K**

**A**


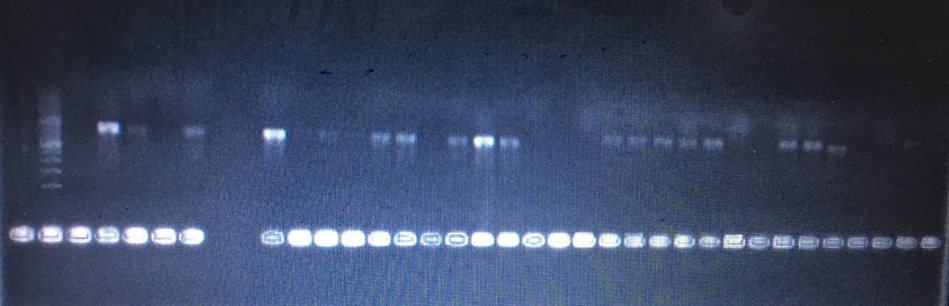


**B**

**Pk**

**Bb**

**Db**

**M**

**K**

**Fig. S1.** Agarose gel images of selected bacteria (A) and yeasts (B) (M: Marker, K: Negative control)


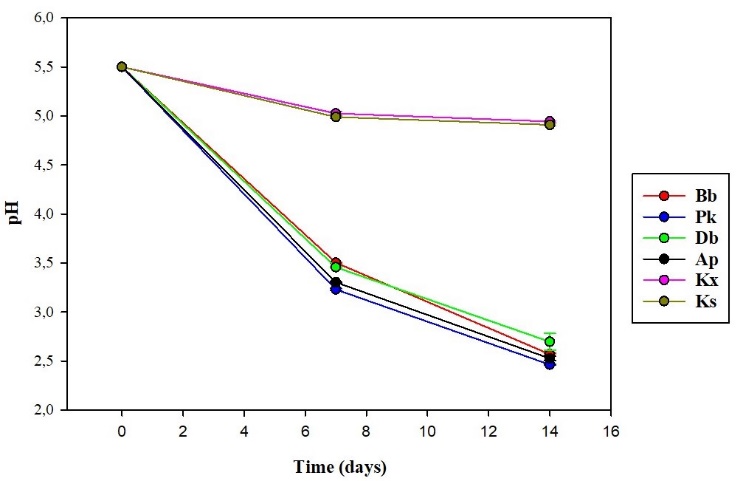

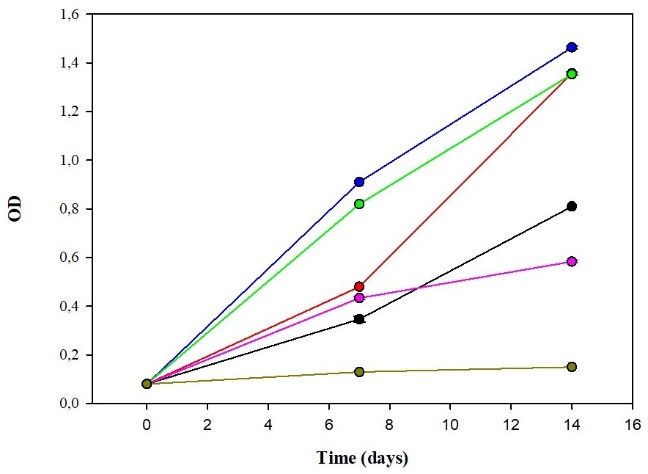


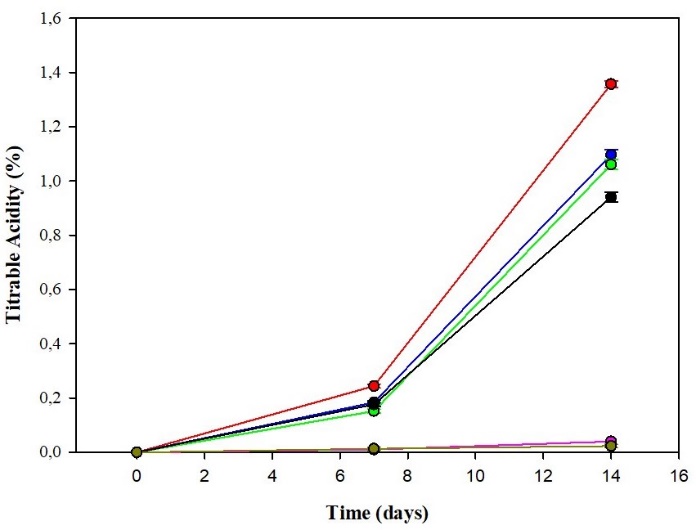

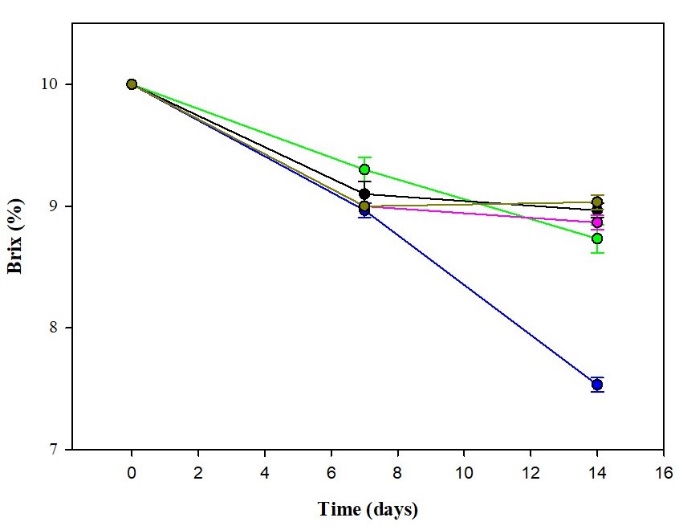

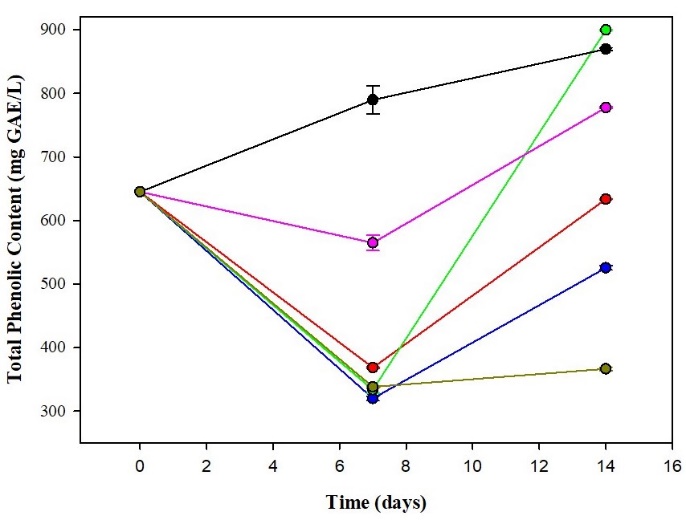

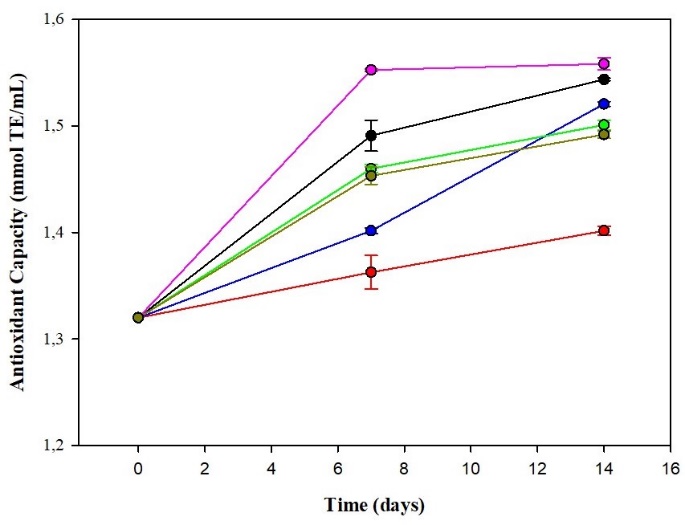


**Fig. S2.** Chemical analysis results at different times of monoculture fermentations (Different letters indicate significant difference in end and start of fermentation, P < 0.05, Pk: *Pichia kudriavzevii*, Bb: *Brettanomyces bruxellensis*, Db: *Dekkera bruxellensis*, Ap: *Acetobacter papayae*, Kx: *Komagataeibacter xylinus*, Ks: *Komagataeibacter saccharivorans*)


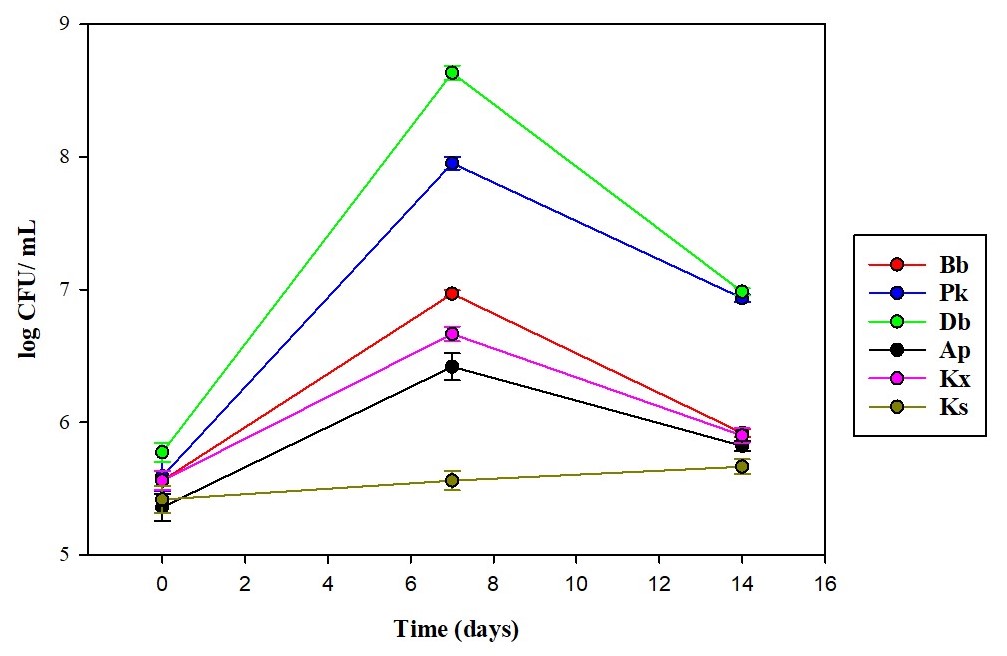


**Fig. S3.** Microbial counts of monoculture fermentations (Different letters show significant difference on different fermentation days of a strain, P < 0.05, Pk: *Pichia kudriavzevii*, Bb: *Brettanomyces bruxellensis*, Db: *Dekkera bruxellensis*, Ap: *Acetobacter papayae*, Kx: *Komagataeibacter xylinus*, Ks: *Komagataeibacter saccharivorans*)


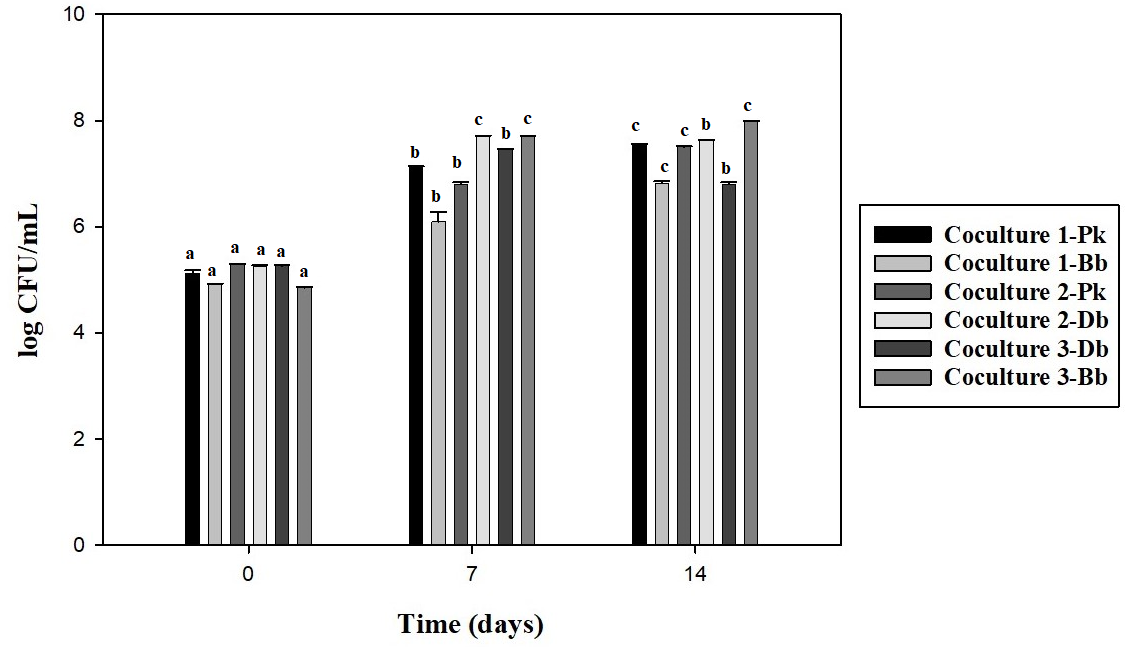


**Fig. S4.** Microbial counts of yeast-yeast fermentations (Different letters show significant difference on different fermentation days of a strain, P < 0.05, Db-Pk: *Dekkera bruxellensis* - *Pichia kudriavzevii*, Bb-Db: *Brettanomyces bruxellensis* - *Dekkera bruxellensis*, Bb-Pk: *Brettanomyces bruxellensis* - *Pichia kudriavzevii*)


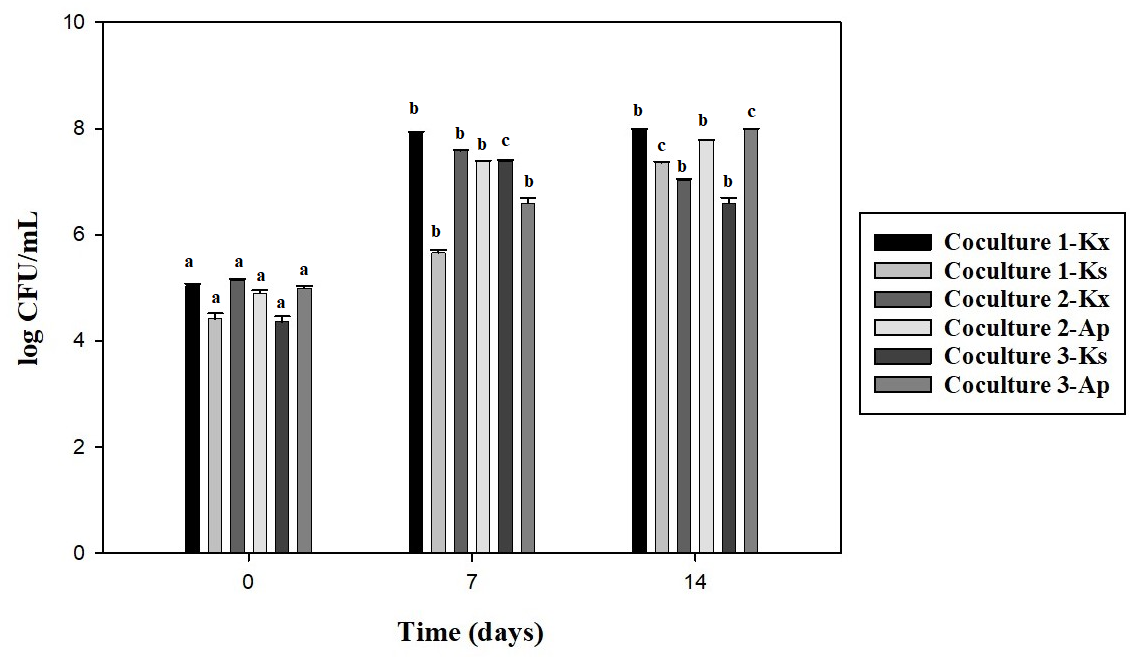


**Fig. S5.** Microbial counts of bacteria-bacteria fermentations (Different letters show significant difference on different fermentation days of a strain, P < 0.05, Kx - Ks: *Komagataeibacter xylinus* - *Komagataeibacter saccharivorans*, Ap - Kx: *Acetobacter papayae* - *Komagataeibacter xylinus*, Ap - Ks: *Acetobacter papayae* - *Komagataeibacter saccharivorans*)


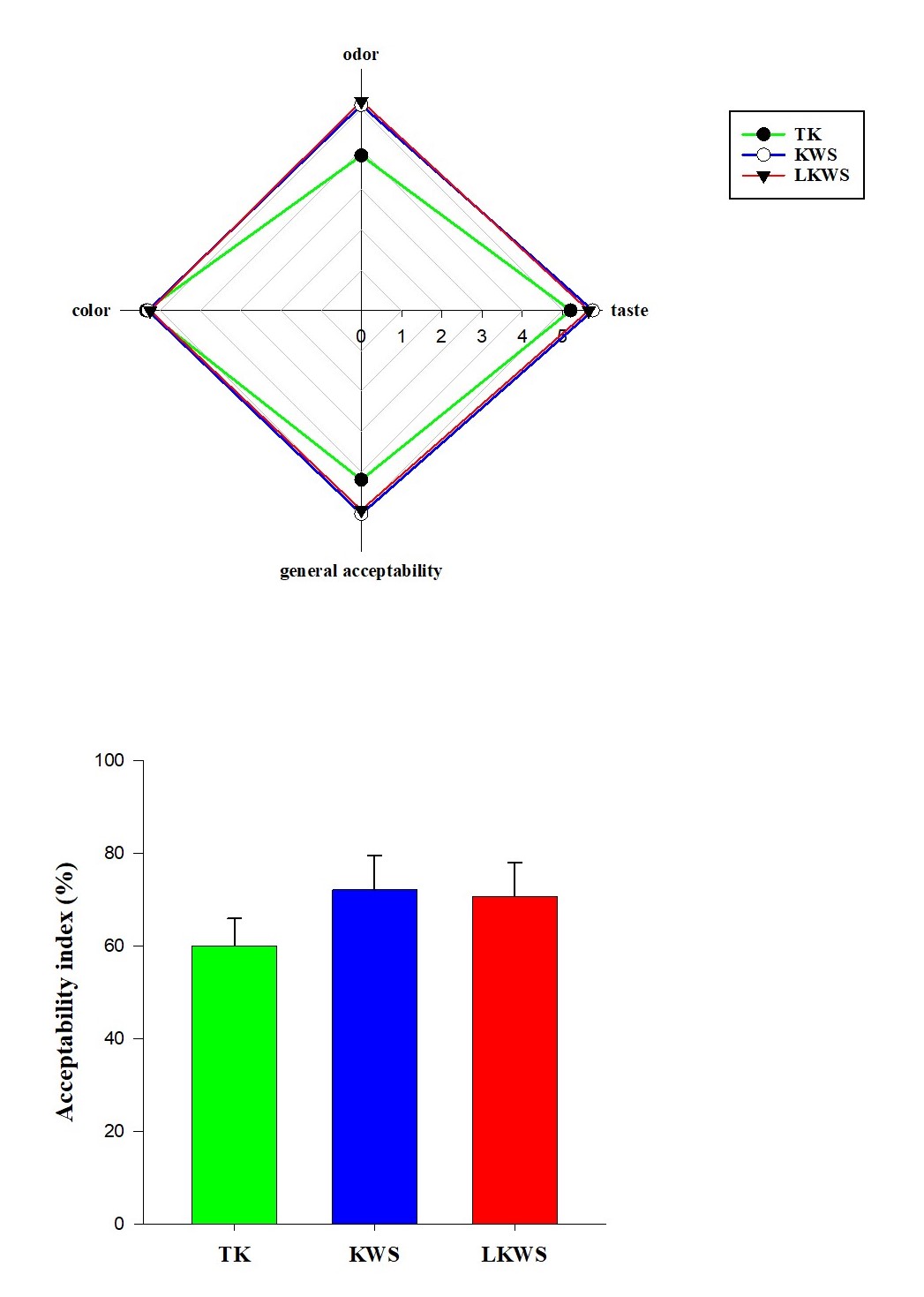


**Fig. S6.** Radar graph and acceptability indexes of kombucha produced with traditional and starter culture (TK: Traditional kombucha, KWS: Kombucha produced with starter culture, LKWS: Kombucha produced with lyophilized starter culture, KI: Acceptability index)

**Table S1** Nucleotide sequences after the sequencing process of selected bacteria and yeasts

***Brettanomyces bruxellensis* CBS:2796 (Bb) - KY103322.1**

GCTCTGATGCATGGCATAGCCCGTGCAGACACGTGGATAAGCAAGGATAAAAATACATTAAATTTATTTAGTTTAGTCAAGAAAGAATTTTAAAACTTTCAACAATGGATCTCTTGGTTCTCGCGTCGATGAAGAGCGCAGCGAATTGCGATACTTAATGTGAATTGCAGATTTTCGTGAATCATCGAGTTCTTGAACGCACATTGCGCCCTCTGGTATTCCGGAGGGCATGCCTGTTTGAGCGTCATTTCCTTCTCACTATTTAGTGGTTATGAGATTACACGAGGGTGTTTTCTTCAAAGGGAAGAGGGGAGTGAGGGGATAATGATTTAAGGTTTCGGCCGTTCATTATTTTTTCTTCTCCCCCAGTTATCAAGTTTGACCTCAAATCAGGTAGGAGGACCCGCTGAACTTAAGCATATCAATAAGCCGGAGGAAAGATCATTACGGATGCTGGGCATAAGCCCGTGCAGACACGTGGATCAGCAAGGATAAAAATACATTAAATTTATTAGTTTAGTCAAGAAAGAATTTTAAAACTTTCAACAATGGATCTTGGTTCTGCGTCGATAAGAGCGCACGAATGCGATACTTAAAGTGAATTGCAATTTCTGAATCACGAGTTCTTGAACGCACATTGCGCCTCTGGTATTCCGGAGGGCATGCCTGTTTGAGCGCTCAGTTCCTCTCCTATGTAGTGGATTATGAGATTACACGAGCGTGTTCTTCTTCAAGAGGAAGAGGGGAGTGAGGGGATAATGATTTAACGTTTCGGCATCATTATATTTTCTCTCCCTTTTAGATTGCACACATCAGTGGATGAACGCCTCACTTAAACGATCATAACCGGGGAGGAGAAAA

***Dekkera bruxellensis* ATCC 200341 (Db) - FJ545249.1**

CTTAGTGTGCTGCGTATGGCGCAGCCGTGCAGACACGTGGATAAGCAAGGATAAAAATACATTAAATTTATTTAGTTTAGTCAAGAAAGAATTTTAAAACTTTCAACAATGGATCTCTTGGTTCTCGCGTCGATGAAGAGCGCAGCGAATTGCGATACTTAATGTGAATTGCAGATTTTCGTGAATCATCGAGTTCTTGAACGCACATTGCGCCCTCTGGTATTCCGGAGGGCATGCCTGTTTGAGCGTCATTTCCTTCTCACTATTTAGTGGTTATGAGATTACACGAGGGTGTTTTCTTCAAAGGGAAGAGGGGAGAGAGAGGAGAAAGAGATTTAAGGTTTCGGCCGTTCATTATTTTTTTCTTCTGCCCCAATTATCAAGTTTGACCTCAAATCAGGGAGGAGGACCCGCTGAACTTAATCATATCAATAAGCGGAGGAAA

***Pichia kudriavzevii* (Pk) - MT781361.1**

GCCGTGCGTTTGCATCTTCCACACGTGCGTGAGCGCAAGCAAAACACGAAAAAACTGTAGTACGAGAGTCAAAACAAACCAAAAAACAAAACTTTCAACAACGGATCTCTTGGTTCTCGCATCGATGAAGAGCGCAGCGAAATGCGATACCTAGTGTGAATTGCAGCCATCGTGAATCATCGAGTTCTTGAACGCACATTGCGCCCTCTGGTATTCCGGAGGGCATGCCTGTTTGAGCGTCGTTTCCTTCTTGCTTGCGAGCAGAAATGGGGGGGCCCTGGCATTGGGGCCGCTCTGAAAAGAAACGTTGCGGGCGAAGCGAACTATGAGTAGGACGCTTGGCCGCCGAACTTAATACATAAGCTCGACCTCAAATCAGGTAGGAATACCCGCTGAACTTAAGCATATCAATAAGCGGAGGAAAAGGATCATTACTGTGATTTAACATCTTCCACACGTGCGTGAGCGCAAGCAAAACACGAAAAAACTGTAGTACGAGAGTCAAAACAAACAAAAAACAAAACTTTCAACAACGGATCTCTTGGTTCTCGCATCGATAAAGCGCAGCGAAATGCGATACTAGTGTGAATTGCAGCTGTGAATCATCAGTTCTTGAACGCACATTGCCTCTGTATTCGGAGGGCATGCTGTTTGAGCGTCGTTTCTTTCTTGCTTGCGAGCAGAAATGGGGGGGTCTGGCATTGGGGCGCTCTGAAAAGAAACGATGCGGGCGAAGCGAACTATGAGTAGGACGCTGGCGGACTAATACATAAGCTCACTCAATCAGGTGGATACCGCTCAACTTAAGCATATCATAGGCGGAGGAA

***Acetobacter papayae* VTH-AH49 (Ap) - LC103256.1**

AGAGTCGCAGCTGTGATGACTGGGCGTCAGGGCGTGTAGGCGGTTTTGACAGTCAGATGTGAAATCCCCGGGCTTAACCTGGGAGCTGCATTTGAGACGTTAAGACTAGAGTGTGAGAGAGGGTTGTGGAATTCCCAGTGTAGAGGTGAAATTCGTAGATATTGGGAAGAACACCGGTGGCGAAGGCGGCAACCTGGCTCATTACTGACGCTGAGGCGCGAAAGCGTGGGGAGCAAACAGGATTAGATACCCTGGTAGTCCACGCTGTAAACGATGTGTGCTAGATGTTGGGTAACTTAGTTACTCAGTGTCGCAGTTAACGCGTTAAGCACACCGCCTGGGGAGTACGGCCGCAAGGTTGAAACTCAAAGGAATTGACGGGGGCCCGCACAAGCGGTGGAGCATGTGGTTTAATTCGAAGCAACGCGCAGAACCTTACCAGGGCTTGAATGTGGAGGATCTAGGCAGAGATGTCTATTTCTTCGGACCTCCCACACAGGTGCTGCATGGCTGTCGTCAGTGGGGTCCCGGGGGGA

***Komagataeibacter xylinus* XJL-02-1 (Kx) - MH511551.1**

GCACGTGCTCGGATGACTGGGCGTAAGGGCGCGTAGGCGGTTTTAACAGTCAGATGTGAAATTCCTGGGCTTAACCTGGGGGCTGCATTTGATACGTTGAGACTAGAGTGTGAGAGAGGGTTGTGGAATTCCCAGTGTAGAGGTGAAATTCGTAGATATTGGGAAGAACACCGGTGGCGAAGGCGGCAACCTGGCTCATTACTGACGCTGAGGCGCGAAAGCGTGGGGAGCAAACAGGATTAGATACCCTGGTAGTCCACGCTGTAAACGATGTGTGCTGGATGTTGGGTGACTTTGTCATTCAGTGTCGTAGTTAACGCGATAAGCACACCGCCTGGGGAGTACGGCCGCAAGGTTGAAACTCAAAGGAATTGACGGGGGCCCGCACAAGCGGTGGAGCATGTGGTTTAATTCGAAGCAACGCGCAGAACCTTACCAGGGCTTGACATGCGGAGGCCGTGTCCAGAGATGGGCATTTCTCGCAAGAGACCTCCAGCACAGGTGCTGCATGGCTGTCGTCAGCGGGGGGCCGGGAGAAA

***Komagataeibacter saccharivorans* strain C6 (Ks) - MT396222.1**

GGACGGCGCTCGGATGACTGGGCGTAAGGGCGCGTAGGCGGTTTTAACAGTCAGATGTGAAATTCCTGGGCTTAACCTGGGGGCTGCATTTGATACGTTGAGACTAGAGTGTGAGAGAGGGTTGTGGAATTCCCAGTGTAGAGGTGAAATTCGTAGATATTGGGAAGAACACCGGTGGCGAAGGCGGCAACCTGGCTCATTACTGACGCTGAGGCGCGAAAGCGTGGGGAGCAAACAGGATTAGATACCCTGGTAGTCCACGCTGTAAACGATGTGTGCTGGATGTTGGGTGACTTTGTCATTCAGTGTCGTAGTTAACGCGATAAGCACACCGCCTGGGGAGTACGGCCGCAAGGTTGAAACTCAAAGGAATTGACGGGGGCCCGCACAAGCGGTGGAGCATGTGGTTTAATTCGAAGCAACGCGCAAAACCTTACCAGGGCTTGACATGCGGAGGCCGTGTCCAGAAGATGGGCATTTCTCGCAAGAAGACCTCCCGCACAGGTTCCTGCATGCTGTAGCGCGGCGAGAGGAGGGGAGAGAGAAA

**Table S2** The amount sugars and acetic acid produced in monoculture fermentations (mean ± standard deviation)

| Strains | | Db | Pk | Bb | Ap | Kx | Ks |
| --- | --- | --- | --- | --- | --- | --- | --- |
| Glucose  (g/L) | **t_0_** | 0±0.0 | 0±0.0 | 0±0.0 | 0±0.0 | 0±0.0 | 0±0.0 |
|  | **t_7_** | 0.81±0.01^b^ | 1.66±0.01^c^ | 2.35±0.01^e^ | 2.03±0.02^d^ | 0.71±0.04^a^ | 0.75±0.01^ab^ |
|  | **t_14_** | 0.97±0.02^b^ | 0±0.0^a^ | 10.71±0.03^e^ | 4.97±0.03^d^ | 0±0.0^a^ | 1.54±0.01^c^ |
| Fructose  (g/L) | **t_0_** | 0±0.0 | 0±0.0 | 0±0.0 | 0±0.0 | 0±0.0 | 0±0.0 |
|  | **t_7_** | 1.55±0.004^c^ | 1.04±0.01^b^ | 1.96±0.01^d^ | 4.44±0.18^d^ | 0±0.0^a^ | 0±0.0^a^ |
|  | **t_14_** | 2.63±0.01^c^ | 0±0.0^a^ | 2.64±0.02^c^ | 19.69±0.25^d^ | 0.97±0.01^b^ | 0.98±0.01^b^ |
| Sucrose  (g/L) | **t_0_** | 101.6±2.09 | 101.6±2.09 | 101.6±2.09 | 101.6±2.09 | 101.6±2.09 | 101.6±2.09 |
|  | **t_7_** | 120.6±0.48^b^ | 123.61±0.4^c^ | 112.93±0.16^a^ | 112.45±0.91^a^ | 177.79±1.97^e^ | 155.33±0.26^d^ |
|  | **t_14_** | 66.12±0.57^b^ | 75.32±0.72^c^ | 11.84±0.16^a^ | 78.14±1.92^c^ | 102.80±0.74^d^ | 120.76±0.52^e^ |
| Acetic acid (mg/L) | **t_0_** | 0±0.0 | 0±0.0 | 0±0.0 | 0±0.0 | 0±0.0 | 0±0.0 |
|  | **t_7_** | 0±0.0 | 0±0.0 | 0±0.0 | 150.90±0.14 | 0±0.0 | 0±0.0 |
|  | **t_14_** | 318.05±0.2^e^ | 0±0.0^a^ | 401.15±0.26^f^ | 315.94±0.38^d^ | 17.68±0.07^c^ | 14.67±0.14^b^ |

t_0_: Fermentatiton day 0, t_7_: Fermentatiton day 7, t_14_: Fermentatiton day 14, different letters in the same row show significant differences (P < 0.05) according to the Tukey test

**Table S3** The amount of sugars and acetic produced by yeast-yeast fermentations (mean ± standard deviation)

| Strains | | Db-Pk | Bb-Db | Bb-Pk |
| --- | --- | --- | --- | --- |
| Glucose  (g/L) | **t_0_** | 0±0.0 | 0±0.0 | 0±0.0 |
|  | **t_7_** | 1.99±0.04^a^ | 8.50±0.02^b^ | 18.88±0.03^c^ |
|  | **t_14_** | 1.22±0.02^a^ | 3.78±0.07^b^ | 30.18±0.03^c^ |
| Fructose  (g/L) | **t_0_** | 0±0.0 | 0±0.0 | 0±0.0 |
|  | **t_7_** | 0.60±0.01^a^ | 0.71±0.03^a^ | 12.52±0.11^b^ |
|  | **t_14_** | 0±0.0^a^ | 2.71±0.02^b^ | 21.84±0.11^c^ |
| Sucrose  (g/L) | **t_0_** | 101.60±2.09 | 101.60±2.09 | 101.60±2.09 |
|  | **t_7_** | 87.25±0.1^c^ | 74.20±0.04^b^ | 19.72±0.24^a^ |
|  | **t_14_** | 81.03±0.25^c^ | 70.04±0.2^b^ | 13.17±0.06^a^ |
| Acetic acid  (mg/L) | **t_0_** | 0±0.0 | 0±0.0 | 0±0.0 |
|  | **t_7_** | 0±0.0 | 0±0.0 | 390.73±0.54 |
|  | **t_14_** | 0±0.0 | 0±0.0 | 211.90±0.16 |

t_0_: Fermentatiton day 0, t_7_: Fermentatiton day 7, t_14_: Fermentatiton day 14, different letters in the same row show significant differences (P < 0.05) according to the Tukey test.

**Table S4** The amount of sugars and acetic acid produced by bacteria fermentations (mean ± standard deviation)

| Strains | | Kx-Ks | Ap-Kx | Ap-Ks |
| --- | --- | --- | --- | --- |
| Glucose  (g/L) | **t_0_** | 0±0.0 | 0±0.0 | 0±0.0 |
|  | **t_7_** | 0.3±0.02^a^ | 2.86±0.02^b^ | 1.99±0.04^a^ |
|  | **t_14_** | 1.0±0.02^a^ | 1.17±0.01^b^ | 1.22±0.02^a^ |
| Fructose  (g/L) | **t_0_** | 0±0.0 | 0±0.0 | 0±0.0 |
|  | **t_7_** | 0.12±0.01^a^ | 2.01±0.01^b^ | 0.60±0.01^a^ |
|  | **t_14_** | 0.82±0.02^a^ | 1.0±0.02^b^ | 0±0.0^a^ |
| Sucrose  (g/L) | **t_0_** | 101.6±2.09 | 101.6±2.09 | 101.6±2.09 |
|  | **t_7_** | 99.05±0.06^c^ | 86.24±0.02^b^ | 87.25±0.1^c^ |
|  | **t_14_** | 73.83±0.04^c^ | 70.4±0.01^b^ | 81.03±0.25^c^ |
| Acetic acid (mg/L) | **t_0_** | 0±0.0 | 0±0.0 | 0±0.0 |
|  | **t_7_** | 0±0.0 | 132.71 | 0±0.0 |
|  | **t_14_** | 0±0.0^a^ | 82.23^b^ | 0±0.0 |

t_0_: Fermentatiton day 0, t_7_: Fermentatiton day 7, t_14_: Fermentatiton day 14, different letters in the same row show significant differences (P < 0.05) according to the Tukey test.
